# Physician, Restore Thyself? The Digital Gap in Physician Well-Being Support

**DOI:** 10.2196/93338

**Published:** 2026-02-27

**Authors:** Jenny Castillo Cato

**Keywords:** physicians, peer support, social support, mental health, physician burnout, well-being, occupational stress, mobile apps, digital health, psychological resilience

**Key Takeaways**

- Physician well-being is a long-standing, complex issue that needs to be better addressed.
- Current digital applications are helpful, but primarily focus on physician self-improvement.
- The digital world is a largely untapped medium for supporting equally critical factors: community, connection, and culture change.

*Jenny Castillo Cato is board-certified in emergency medicine and has worked as an attending physician for over 20 years. She is an advocate for physician well-being and has worked on physician well-being initiatives in New York and across the United States.*

In March 2020, the COVID-19 pandemic hit New York City like a tornado. The flood of patients coming to the emergency departments of the five boroughs was beyond imagination. Staff were getting sick, supplies were running out, and no treatment had been developed for this brand-new virus.

As physicians, we had to make hard decisions. Should I put the 80-year-old man who was barely responsive and hypoxic on the last ventilator in the hospital? Or should I keep the ventilator for the next patient who may be younger and more likely to withstand the onslaught of this deadly virus? Almost 6 years later, those memories still haunt me.

## Unspoken Burden

Physicians have long been expected to rise to these kinds of occasions and to perform at a high level across many challenging circumstances, and we do. There is, however, an emotional toll on physician well-being, one that we are frequently expected to carry alone. This issue is not new. Though often hidden out of fear of discovery and shame, it has persisted throughout the centuries. In the 1860s, Dr James Still wrote in his memoirs that he often felt lonely, with “no one with whom to hold conference or to cheer or comfort me when depressed with difficult cases” [1]. This mentality has continued through the ages, was highlighted during the COVID-19 pandemic, and remains a pressing concern today.

Physicians’ mental health is a crucial aspect of well-being—ours and, ultimately, our patients’—yet the stigma surrounding mental health assistance has led to dire consequences. The American Medical Association reports that physicians are 82% more likely to experience burnout than those in other fields [2]. Furthermore, in the United States, between 300 and 400 physicians die by suicide each year, and the suicide rate for female physicians is 250% to 400% higher than for female workers in other professions [3]. Estimates vary and tend to be higher in the United States, but one study suggests that, across countries, physicians die by suicide, on average, 44% more often than expected compared to the general population [4]. An increase in risk factors (such as administrative burden and excessive job demands) and a decline in protective factors (such as autonomy and peer-based support) may play a role [5].

Foundations like the Dr. Lorna Breen Hero’s Foundation—named after my friend and colleague who died by suicide in April 2020—were created to advocate for change [6]. Nonetheless, progress remains slow and challenging due to factors such as physician personality traits, stigma within the medical community, and entrenched norms, including mandatory mental health questions on licensure applications that perpetuate the idea that mental illness equates to weakness or incompetence [7].

## Persistent Gaps in Support

To support physician mental health and well-being, the medical community promotes self-care such as mindfulness, exercise, better sleep habits, and nutrition.

Many organizations, such as the American College of Physicians and the American Academy of Allergy, Asthma, and Immunology, offer useful well-being resources for this kind of self-improvement. The resources highlight applications such as Headspace, a meditation-based app; My Well-Being Index, an anonymous evaluation tool from the Mayo Clinic; Provider Resilience, a self-assessment tool for compassion fatigue and burnout; and T2 Mood Tracker, an app that monitors anxiety, depression, posttraumatic stress disorder, and general well-being [8,9]. There are also a few specialty-specific applications like the Surgeon’s Masters, which focus on physical wellness with the 7-minute workout; Sleepo, a sleep tracker; and WaterMinder, a way to monitor hydration [10].

But while informative and helpful, these applications do not fully address the complex issue of physician well-being. The COVID-19 pandemic highlighted that well-being could not be addressed solely at the individual level but that significant changes must be made in clinical environments as well. Vast organizational norms need to be altered to transform physician well-being; it requires a cultural, political, and environmental shift.

There is also no one right answer for supporting physician well-being; it cannot be accomplished through a “one size fits all” approach. Each medical specialty has different challenges and needs. For example, what is important and impacts an orthopedic surgeon’s daily work-life is very different from what matters to a family medicine physician seeing patients in a clinic. The physical stress, mental toll, and teamwork required during an 8-hour surgery affect the surgeon differently than a physician who manages 40 patients who are chronically ill every 15 minutes for 8 hours.

In short, persistent gaps remain in the resources available for physicians.

## New Solutions

Like Dr Still noted in the 1860s, physicians need community, support, and camaraderie. This is particularly pressing as many medical institutions—especially in New York City, where space is a premium—have opted to use physicians’ lounges for administrative purposes, leading to less direct physician contact across specialties.

A new app called Roon is trying to address this issue. Though Roon began as a video-based question and answer platform of health issues for patients, the final product created a “physicians-only knowledge network” providing a “digital doctor’s lounge where wisdom of one becomes the resource of many” [11]. Roon and other programs like PeerRxMed, a free health care provider peer-to-peer program [12], are beginning to address the complex nature of physician well-being by facilitating community, peer support, and camaraderie. Beyond these tools, however, solutions in the digital space have yet to be fully tapped into.

## A Call to Action

More resources are needed to support physician mental health and well-being, particularly resources that go beyond self-care and self-improvement.

New applications need to be created with a collaborative mindset. Clinicians must work with programmers and graphic designers to create content that is relevant and specific to each specialty. Combining directed self-care options, team building, leadership skills, and peer support into an easy-to-use, focused application would be incredibly valuable for the newer generations entering medicine.

The consequences of *not* developing these tools include and extend beyond physicians: without a healthy medical workforce, everyone would be affected. While we love our profession, physicians need resources to help us not only put on our own masks first but also support one another and share the weight so that we can more effectively care for patients.

The digital world remains an untapped medium for building physician community and culture change. Let us act now to harness its power to help physicians holistically cultivate better health, positive work-life integration, and institutional changes, creating an environment that allows longer, happier careers.
